# Personal Care Products and Incident Hypertension: Prospective Cohort Study of U.S. Women

**DOI:** 10.64898/2026.05.18.26353536

**Published:** 2026-05-21

**Authors:** Jungeun Lim, Che-Jung Chang, Alexandra White, Gabriel Goodney, Hantao Wang, Jungnam Joo, Véronique L Roger, Dale P Sandler, Jason YY Wong

## Abstract

Background: Over half of U.S. women have hypertension, a strong but modifiable risk factor for cardiovascular diseases. Personal care products (PCPs) are widely used in daily life and contain endocrine disrupting chemicals that can alter hormonal regulation of blood pressure. However, the relationship between PCPs and hypertension has not been well studied. We investigated whether patterns of PCP use were associated with incident hypertension in a large prospective cohort study of U.S. women. Methods: Sister Study participants were recruited in 2003-2009 and followed until September 30, 2021. Usage frequency of 41 PCPs in the 12 months before baseline was self-reported. Latent class analyses identified groups with similar PCP use patterns ("infrequent," "moderate," or "frequent"). At baseline, we excluded women with prevalent hypertension, antihypertensive medication users, or those missing hypertension status. Multivariable Cox regression was used to estimate associations between PCP use and incident self-reported hypertension. Results: During a mean follow-up of 11.4 years, 10,099 women developed hypertension. Frequent PCP use was associated with higher hypertension risk [HR=1.08 (95% CI: 1.03, 1.13); p-trend=0.003], with a 4.1% population attributable risk. Frequent users of beauty products had higher risk than infrequent users [HR=1.11 (95% CI: 1.05, 1.16)]. Moderate and frequent users of hygiene products also had increased risk [HR=1.07 (95% CI: 1.01, 1.13); HR=1.13 (95% CI: 1.08, 1.19)]. Conclusions: Frequent PCP use, especially beauty and hygiene products, was associated with incident hypertension. Our findings implicate everyday chemicals as modifiable cardiovascular risk factors and highlight the need to identify pathogenic components in widely used consumer products.

## Full Text Availability

The license terms selected by the author(s) for this preprint version do not permit archiving in PMC. The full text is available from the preprint server.

