# Supplementary material for: Personal Care Products and Incident Hypertension: Prospective Cohort Study of U.S. Women
Source: medRxiv. 2026 Jun 8:2026.05.18.26353536. Originally published 2026 May 21. Preprint. [Version 2] doi: 10.64898/2026.05.18.26353536 (PMC13228694; doi:10.64898/2026.05.18.26353536)
Supplement: Supplement 1 [file media-1.pdf]

eTable 1. Thirty-three products selected by random survival forest.

Ranking	Products	Product groups
1	Hair food	Hair products
2	Artificial nails (else)	Beauty products
3	Face cream	Skincare products
4	Talc (vaginal)	Hygiene products
5	Artificial nails	Beauty products
6	Douche	Hygiene products
7	Nail polish remover	Beauty products
8	Body lotion	Skincare products
9	Lip Moisturizer	Skincare products
10	Minoxidil	Hair products
11	Conditioner	Hair products
12	Lipstick	Beauty products
13	Hand lotion	Skincare products
14	Perfume	Beauty products
15	Mascara	Beauty products
16	Skin lightener	Skincare products
17	Blush	Beauty products
18	Nail polish	Beauty products
19	Talc (other)	Hygiene products
20	Deodorant	Hygiene products
21	Talc (under arms)	Hygiene products
22	Pomade	Hair products
23	Eye liner	Beauty products
24	Hair styling gel	Hair products
25	Hair spray	Hair products
26	Age spot lightener	Skincare products
27	Baby oil	Skincare products
28	Anti-aging product	Skincare products
29	Self-tanning product	Skincare products
30	Foundation	Beauty products
31	Foot cream	Skincare products
32	Cleansing cream	Skincare products
33	Bath gel	Hygiene products

eFigure 1. Variable Importance Plot of random survival forest

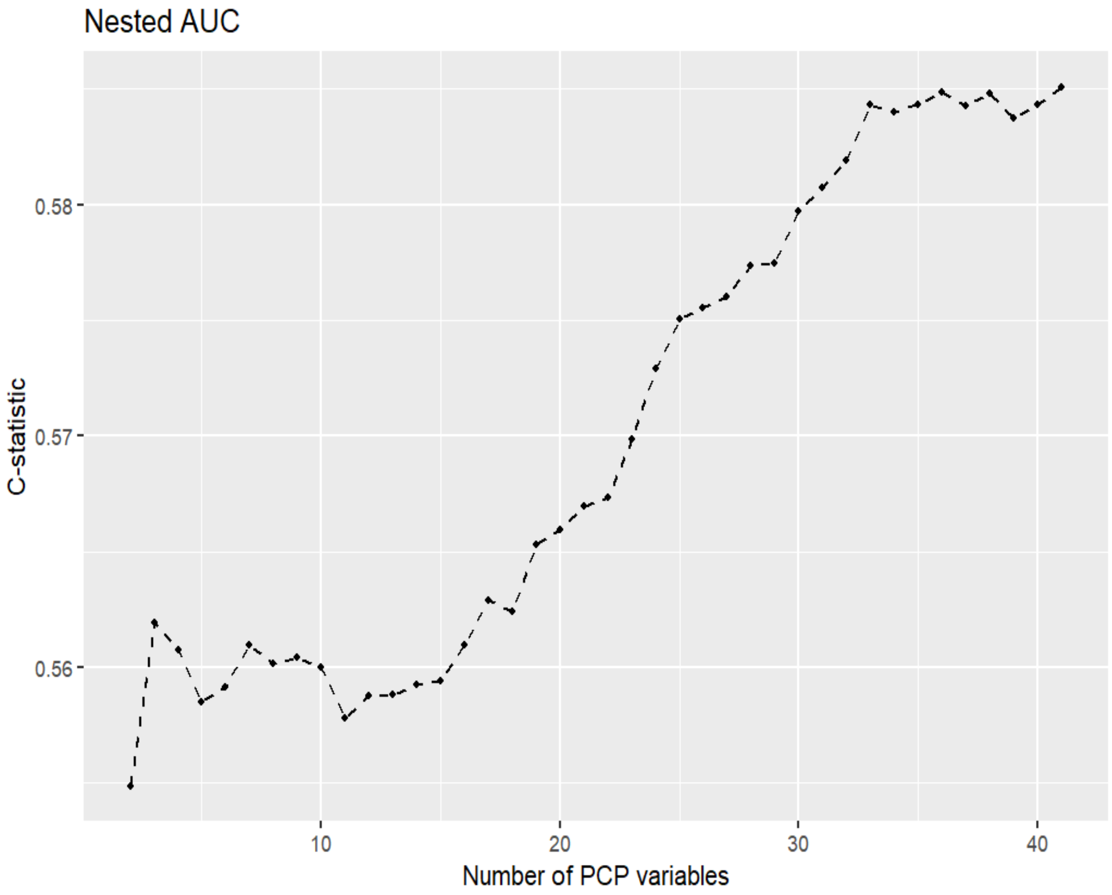

eTable 2. Summary of information for selecting number of latent classes of overall personal care product use [a]

Number of Latent Classes	G^2	AIC	BIC	CAIC	a-BIC
2	1359975.66	1360505.66	1362715.67	1362980.67	1361873.50
3	1325381.50	1326177.50	1329496.68	1329894.68	1328231.84
4	1310596.15	1311658.15	1316086.50	1316617.50	1314398.99
5	1298709.27	1300037.27	1305574.80	1306238.80	1303464.61

G=Likelihood Ratio Chi-square; AIC=Akaike information criterion; BIC=Bayesian information criterion; CAIC=Consistent Akaike Information Criterion; a-BIC=sample size-adjusted Bayesian information criterion

[a] It is common for AIC to continue decreasing as the number of classes increases, which can lead to over-extraction. While BIC is generally more reliable, in cases of very large sample sizes, the penalty term can become too small relative to the log-likelihood, leading to over-extraction. Therefore, we considered not only the significant decrease in AIC and BIC values compared to the previous model, but also the substantive theory and interpretability to enhance the validity and interpretability of the model.

eTable 3. Latent class descriptions of overall personal care product use[a]

Labels of latent classes	Class description
A. Infrequent users	Likely to have infrequent use of 33 personal care products (hair food, artificial nails (someone else), face cream, talcum powder applied vaginal area, artificial nails, douche, nail polish remover, body lotion, lip moisturizer, minoxidil, conditioner, lipstick, hand lotion, perfume, mascara, skin lightener, blush, nail polish, talcum powder applied other area, deodorant, talcum powder applied under arms area, pomade, eye liner, hair styling gel, hair spray, age spot lightener, baby oil, anti-aging product, self-tanning product, foundation, foot cream, cleansing cream, and bath gel)
B. Moderate users	Likely to have moderate use of hair food, talcum powder applied vaginal area, artificial nails, douche, body lotion, lip moisturizer, minoxidil, conditioner, lipstick, hand lotion, perfume, mascara, skin lightener, blush, nail polish, talcum powder applied other area, deodorant, talcum powder applied under arms area, pomade, eye liner, hair styling gel, hair spray, age spot lightener, baby oil, anti-aging product, self-tanning product, foundation, foot cream, cleansing cream, and bath gel, and likely to have frequent use of artificial nails (someone else), face cream, and nail polish remover
C. Frequent users	Likely to have frequent use of hair food, talcum powder applied vaginal area, artificial nails, douche, body lotion, lip moisturizer, minoxidil, conditioner, lipstick, hand lotion, perfume, mascara, skin lightener, blush, nail polish, talcum powder applied other area, deodorant, talcum powder applied under arms area, pomade, eye liner, hair styling gel, hair spray, age spot lightener, baby oil, anti-aging product, self-tanning product, foundation, foot cream, cleansing cream, and bath gel, and likely to have moderate use of artificial nails (someone else), face cream, and nail polish remover

[a] Class labels and descriptions are based on likely item response probabilities for each product, but all responses for individual women may not fit these parameters; each class is described relative to the other classes in each product category (Taylor etal.2017).

eTable 4. Overall personal care product use and risk of hypertension stratified by race, menopausal status, and annual household income[a]

	Infrequent users	Moderate users	Frequent users	P-trend	P for interaction[b]
	HR (95%CI)	HR (95%CI)	HR (95%CI)		
Race and ethnicity					
White	1 (Ref.)	1.001 (0.95-1.06)	1.08 (1.03-1.14)	0.002	0.167
Black	1 (Ref.)	1.13 (0.96-1.34)	1.21 (0.97-1.50)	0.082	
Menopausal status					
Premenopausal	1 (Ref.)	0.997 (0.91-1.09)	1.09 (1.001-1.19)	0.034	0.937
Postmenopausal	1 (Ref.)	1.03 (0.97-1.10)	1.07 (1.01-1.13)	0.033	
Annual Household Income					
Less than $50,000	1 (Ref.)	1.08 (0.98-1.19)	1.12 (1.02-1.24)	0.021	0.311
$50,000 to $99,999	1 (Ref.)	0.98 (0.91-1.07)	1.06 (0.98-1.15)	0.097	
More than $100,000	1 (Ref.)	0.99 (0.90-1.09)	1.04 (0.95-1.14)	0.343	
Obesity Status					
Body mass index<30kg/m^2	1 (Ref.)	1.01 (0.95-1.07)	1.07 (1.01-1.14)	0.011	0.723
30kg/m^2≤ Body mass index	1 (Ref.)	1.002 (0.92-1.10)	1.06 (0.97-1.17)	0.233	

HR=Hazard ratios; CI=95% confidence intervals; Ref.=reference group

[a] Models accounted for age as the timescale. In addition, models were adjusted for race/ethnicity (non-Hispanic Black, non-Hispanic White, Hispanic, other, or missing), body mass index (continuous, kg/m^2), education level (high school or less, some college, college and above, or missing), annual household income (<$50,000, $50,000-<$100,000, ≥$100,000), menopausal status at enrollment (premenopausal, postmenopausal, or missing), smoking pack-year (continuous, pack-year), alcohol consumption (never or past, current <1 drink, current ≥1 drinks, or missing), and physical activity (continuous, hours per week)

[b] Interactions were assessed through likelihood ratio tests by comparisons of Cox models with and without the cross-product of each factor and latent class.

eTable 5. Association between personal care products and risk of hypertension, additionally adjusting for family history of hypertension[a,b]

	Infrequent users	Moderate users	Frequent users	Frequent users of talcum powder	P-trend
Product groups	HR (95% CI)	HR (95% CI)	HR (95% CI)	HR (95% CI)	
Beauty products	1 (Ref.)	0.999 (0.95-1.06)	1.09 (1.04-1.15)	−	<0.001
Everyday hair products	1 (Ref.)	0.98 (0.89-1.09)	1.001 (0.90-1.12)	−	0.603
Hygiene products	1 (Ref.)	1.06 (1.00-1.13)	1.12 (1.06-1.18)	1.18 (1.10-1.27)	<0.001
Skincare products	1 (Ref.)	1.02 (0.96-1.08)	1.00 (0.95-1.05)	−	0.939

HR=Hazard ratios; CI=95% confidence intervals; Ref.=reference group

[a] Numbers of total subjects and hypertension events are for women with complete data for each product class and covariates only

[b] Models accounted for age as the timescale. In addition, models were adjusted for race/ethnicity (non-Hispanic Black, non-Hispanic White, Hispanic, other, or missing), body mass index (continuous, kg/m^2), education level (high school or less, some college, college and above, or missing), annual household income (<$50,000, $50,000-<$100,000, ≥$100,000), menopausal status at enrollment (premenopausal, postmenopausal, or missing), smoking pack-year (continuous, pack-year), alcohol consumption (never or past, current <1 drink, current ≥1 drinks, or missing), physical activity (continuous, hours per week), and family history (biological parents) of hypertension

eTable 6. Association between frequency of individual beauty product use in the past 12 months before baseline and risk of hypertension[b]

	Did not use	Less than once a month	1-3 times per month	1-5 times per week	More than 5 times per week	P-trend	FDR adjusted P-trend[c]
	HR (95%CI)	HR (95%CI)	HR (95%CI)	HR (95%CI)	HR (95%CI)		
Blush or rouge	1.00 (Ref.)	1.00 (0.94-1.08)	0.98 (0.91-1.06)	1.02 (0.96-1.08)	1.05 (0.998-1.11)	0.047	0.077
Eye liner	1.00 (Ref.)	0.99 (0.92-1.06)	1.01 (0.93-1.09)	0.97 (0.91-1.02)	1.03 (0.97-1.08)	0.667	0.667
Eye shadow	1.00 (Ref.)	1.02 (0.96-1.08)	1.02 (0.95-1.09)	1.06 (0.998-1.12)	1.11 (1.05-1.18) [a]	<0.001	<0.001
Foundation makeup	1.00 (Ref.)	1.05 (0.97-1.12)	1.02 (0.95-1.11)	1.08 (1.02-1.14)[a]	1.12 (1.06-1.18) [a]	<0.001	<0.001
Lipstick	1.00 (Ref.)	0.98 (0.90-1.06)	0.98(0.89-1.05)	1.05 (0.98-1.12)	1.08 (1.01-1.15) [a]	0.001	0.002
Eye mascara	1.00 (Ref.)	0.99 (0.92-1.06)	1.05 (0.97-1.13)	1.04 (0.97-1.10)	1.07 (1.02-1.13) [a]	0.004	0.007
Perfume or cologne	1.00 (Ref.)	1.06 (0.99-1.13)	1.07 (1.00-1.14) [a]	1.12 (1.06-1.19)[a]	1.18 (1.10-1.25) [a]	<0.001	<0.001
Makeup remover	1.00 (Ref.)	0.99 (0.93-1.06)	1.03 (0.95-1.13)	1.07 (1.003-1.15)[a]	1.10 (1.03-1.16) [a]	0.001	0.002
Artificial nails/fill-ins someone else	1.00 (Ref.)	1.26 (1.02-1.56) [a]	1.21 (0.89-1.65)	0.63 (0.16-2.53)	-	0.0890	0.129
Artificial nails or fill-ins	1.00 (Ref.)	1.13 (1.04-1.23) [a]	1.17 (1.09-1.25) [a]	1.11 (0.70-1.77)	0.74 (0.35-1.55)	<0.001	<0.001
Cuticle cream	1.00 (Ref.)	1.01 (0.96-1.05)	1.06 (1.00-1.13) [a]	1.04 (0.93-1.17)	0.99 (0.76-1.28)	0.112	0.146
Nail polish	1.00 (Ref.)	1.04 (0.98-1.10)	1.05 (0.99-1.11)	1.01 (0.92-1.10)	1.09 (0.91-1.32)	0.326	0.385
Nail polish remover	1.00 (Ref.)	1.03 (0.97-1.09)	1.04 (0.98-1.10)	0.96 (0.87-1.05)	1.40 (0.94-2.08)	0.666	0.667

HR=Hazard ratios; CI=95% confidence intervals; Ref.=reference group

[a] P values are less than 0.05.

[b] Models accounted for age as the timescale. In addition, models were adjusted for race/ethnicity (non-Hispanic Black, non-Hispanic White, Hispanic, other, or missing), body mass index (continuous, kg/m^2), education level (high school or less, some college, college and above, or missing), annual household income (<\$50,000, \$50,000-<\$100,000, ≥\$100,000), menopausal status at enrollment (premenopausal, postmenopausal, or missing), smoking pack-year (continuous, pack-year), alcohol consumption (never or past, current <1 drink, current ≥1 drinks, or missing), and physical activity (continuous, hours per week).

[c] The false discovery rate (FDR) adjustment was used to correct for multiple testing.

eTable 7. Association between frequency of individual everyday hair product use in the past 12 months before baseline and risk of hypertension[b]

	Did not use	Less than once a month	1-3 times per month	1-5 times per week	More than 5 times per week	P-trend	FDR adjusted P-trend[c]
	HR (95%CI)	HR (95%CI)	HR (95%CI)	HR (95%CI)	HR (95%CI)		
Hair conditioner/rinse	1.00 (Ref.)	0.94 (0.85-1.03)	0.92 (0.84-1.00)[a]	0.96 (0.90-1.02)	0.95 (0.89-1.02)	0.477	0.835
Hair food	1.00 (Ref.)	1.07 (0.95-1.21)	1.03 (0.88-1.22)	0.94 (0.72-1.22)	0.95 (0.53-1.37)	0.966	0.966
Hair spray	1.00 (Ref.)	1.03 (0.97-1.10)	1.08 (1.01-1.16)[a]	1.08 (1.02-1.14)[a]	1.14 (1.07-1.20)[a]	<0.001	<0.001
Hair styling gel/mousse	1.00 (Ref.)	1.03 (0.96-1.10)	0.97 (0.91-1.04)	1.06 (1.003-1.12)[a]	1.01 (0.95-1.08)	0.258	0.602
Minoxidil or Rogaine	1.00 (Ref.)	1.10 (0.79-1.54)	0.91 (0.60-1.38)	1.36 (0.99-1.87)	0.86 (0.66-1.12)	0.846	0.966
Pomade or hair grease	1.00 (Ref.)	1.02 (0.92-1.13)	1.02 (0.92-1.13)	0.95 (0.86-1.06)	0.79 (0.66-0.96)	0.076	0.267
Shampoo	1.00 (Ref.)	1.00 (0.51-1.97)	0.97 (0.52-1.82)	0.94 (0.51-1.75)	0.96 (0.52-1.79)	0.680	0.952

HR=Hazard ratios; CI=95% confidence intervals; Ref.=reference group

[a] P values are less than 0.05.

[b] Models accounted for age as the timescale. In addition, models were adjusted for race/ethnicity (non-Hispanic Black, non-Hispanic White, Hispanic, other, or missing), body mass index (continuous, kg/m^2), education level (high school or less, some college, college and above, or missing), annual household income (<\$50,000, \$50,000-<\$100,000, ≥\$100,000), menopausal status at enrollment (premenopausal, postmenopausal, or missing), smoking pack-year (continuous, pack-year), alcohol consumption (never or past, current <1 drink, current ≥1 drinks, or missing), and physical activity (continuous, hours per week).

[c] The false discovery rate (FDR) adjustment was used to correct for multiple testing.

eTable 8. Association between frequency of individual hygiene product use in the past 12 months before baseline and risk of hypertension[b]

	Did not use	Less than once a month	1-3 times per month	1-5 times per week	More than 5 times per week	P-trend	FDR adjusted P-trend[c]
	HR (95%CI)	HR (95%CI)	HR (95%CI)	HR (95%CI)	HR (95%CI)		
Bath or shower gel	1.00 (Ref.)	1.04 (0.98-1.10)	1.07 (1.01-1.15) [a]	1.05 (0.99-1.11)	1.10 (1.04-1.16) [a]	0.0015	0.002
Deodorant and/or antiperspirant	1.00 (Ref.)	1.05 (0.87-1.27)	0.88 (0.74-1.05)	1.13 (0.995-1.28)	1.27 (1.14-1.42) [a]	<0.001	<0.001
Douche	1.00 (Ref.)	1.11(1.04-1.19) [a]	1.26 (1.11-1.42) [a]	1.11 (0.78-1.59)	1.29 (0.61-2.71)	<0.001	<0.001
Mouthwash/rinse	1.00 (Ref.)	1.03 (0.97-1.09)	1.07(1.01-1.14) [a]	1.07 (1.01-1.14) [a]	1.11 (1.04-1.18) [a]	<0.001	0.001
Shaving creams or gels	1.00 (Ref.)	1.07 (1.01-1.14) [a]	1.11 (1.05-1.17) [a]	1.05 (0.99-1.11)	1.17 (1.02-1.34) [a]	<0.001	0.001
Talcum powder under arms	1.00 (Ref.)	1.00 (0.92-1.07)	1.16 (1.04-1.28) [a]	1.15 (1.03-1.30) [a]	1.05 (0.92-1.20)	0.007	0.008
Talcum powder on vaginal area	1.00 (Ref.)	1.07 (0.99-1.16)	1.20 (1.08-1.33) [a]	1.15 (1.02-1.30) [a]	1.03 (0.90 -1.19)	0.002	0.002
Talcum powder to other areas	1.00 (Ref.)	0.99 (0.94-1.05)	1.01 (0.94-1.08)	1.03 (0.95-1.12)	1.13 (1.03-1.24) [a]	0.032	0.032

HR=Hazard ratios; CI=95% confidence intervals; Ref.=reference group

[a] P values are less than 0.05.

[b] Models accounted for age as the timescale. In addition, models were adjusted for race/ethnicity (non-Hispanic Black, non-Hispanic White, Hispanic, other, or missing), body mass index (continuous, kg/m^2), education level (high school or less, some college, college and above, or missing), annual household income (<\$50,000, \$50,000-<\$100,000, ≥\$100,000), menopausal status at enrollment (premenopausal, postmenopausal, or missing), smoking pack-year (continuous, pack-year), alcohol consumption (never or past, current <1 drink, current ≥1 drinks, or missing), and physical activity (continuous, hours per week).

[c] The false discovery rate (FDR) adjustment was used to correct for multiple testing.

eTable 9. Association between frequency of individual skincare product use in the past 12 months before baseline and risk of hypertension[b]

	Did not use	Less than once a month	1-3 times per month	1-5 times per week	More than 5 times per week	P-trend	FDR adjusted P-trend[c]
	HR (95%CI)	HR (95%CI)	HR (95%CI)	HR (95%CI)	HR (95%CI)		
Anti-aging/wrinkle products	1.00 (Ref.)	1.04 (0.97-1.11)	1.08 (1.002-1.16) [a]	1.03 (0.97-1.09)	1.04 (0.99-1.09)	0.143	0.316
Age spot lightener	1.00 (Ref.)	1.12 (1.02-1.22) [a]	0.94 (0.83-1.06)	1.07 (0.95-1.19)	1.06 (0.93-1.20)	0.215	0.376
Baby oil/mineral-based oils	1.00 (Ref.)	1.04 (0.97-1.10)	1.11 (1.02-1.20) [a]	1.00 (0.91-1.11)	1.07 (0.96-1.19)	0.061	0.284
Blemish/acne products	1.00 (Ref.)	1.04 (0.97-1.10)	1.07 (0.98-1.16)	0.98 (0.88-1.08)	1.00 (0.90-1.12)	0.631	0.736
Body lotions or creams	1.00 (Ref.)	1.03 (0.92-1.16)	0.97 (0.87-1.07)	0.98 (0.90-1.08)	0.97 (0.89-1.06)	0.263	0.409
Cleansing cream	1.00 (Ref.)	1.02 (0.95-1.10)	1.03 (0.95-1.11)	0.98 (0.92-1.05)	0.99 (0.94-1.04)	0.487	0.682
Face creams/moisturizers	1.00 (Ref.)	1.05 (0.94-1.18)	0.97 (0.88-1.08)	0.96 (0.89-1.04)	0.96 (0.90-1.03)	0.102	0.316
Facial masks	1.00 (Ref.)	0.99 (0.95-1.04)	1.01 (0.93-1.09)	1.03 (0.87-1.23)	0.87 (0.56-1.36)	0.921	0.947
Foot creams or moisturizers	1.00 (Ref.)	1.02 (0.96-1.08)	1.05 (0.99-1.11)	1.04 (0.98-1.11)	1.09 (1.02-1.17) [a]	0.007	0.094
Hand lotions or creams	1.00 (Ref.)	1.12 (0.94-1.33)	1.05 (0.90-1.24)	1.06 (0.91-1.23)	1.303 (0.88-1.19)	0.158	0.316
Lip moisturizers	1.00 (Ref.)	1.01 (0.94-1.09)	0.97 (0.90-1.04)	0.95 (0.89-1.02)	0.95 (0.89-1.02)	0.037	0.262
Petroleum jelly	1.00 (Ref.)	0.96 (0.91-1.02)	1.01 (0.93-1.09)	0.93 (0.84-1.02)	1.06 (0.97-1.16)	0.947	0.947
Skin lighteners	1.00 (Ref.)	1.14 (0.99-1.32)	1.00 (0.83-1.20)	0.97 (0.81-1.17)	0.90 (0.73-1.11)	0.606	0.736
Self-tanning products	1.00 (Ref.)	1.04 (0.99-1.10)	1.06 (0.98-1.14)	1.05 (0.95-1.17)	0.92 (0.69-1.23)	0.115	0.316

HR=Hazard ratios; CI=95% confidence intervals; Ref.=reference group

[a] P values are less than 0.05.

[b] Models accounted for age as the timescale. In addition, models were adjusted for race/ethnicity (non-Hispanic Black, non-Hispanic White, Hispanic, other, or missing), body mass index (continuous, kg/m^2), education level (high school or less, some college, college and above, or missing), annual household income (<$50,000, $50,000-<$100,000, ≥$100,000), menopausal status at enrollment (premenopausal, postmenopausal, or missing), smoking pack-year (continuous, pack-year), alcohol consumption (never or past, current <1 drink, current ≥1 drinks, or missing), and physical activity (continuous, hours per week).

[c] The false discovery rate (FDR) adjustment was used to correct for multiple testing.
